# Terahertz Time‐Domain Spectroscopy as a Probe of Skin Viscoelasticity

**DOI:** 10.1111/srt.70402

**Published:** 2026-10-31

**Authors:** Benjamin George Page, Xiangyu Guo, Rayko Ivanov Stantchev, Joseph Hardwicke, Emma Pickwell‐MacPherson

**Affiliations:** ^1^ Department of Physics University of Warwick Coventry UK; ^2^ Department of Physics National Sun Yat‐Sen University Kaohsiung Taiwan; ^3^ Medical School University of Warwick Coventry UK; ^4^ Institute of Applied & Translational Technologies in Surgery (IATTS) University Hospitals Coventry and Warwickshire Coventry UK

**Keywords:** ageing, in vivo, skin, spectroscopy, terahertz, viscoelasticity

## Abstract

In both a cosmetic and clinical setting, there is a strong demand for the development of quantitative methods to assess skin condition and monitor treatment progress. Terahertz (THz) light has shown promise for non‐invasive skin evaluation, in particular for classifying between diseased and healthy tissue based on the differences in dielectric properties. Mechanical properties of the skin are also useful biomarkers for clinicians and cosmeticians, and are typically measured using suction or torsion‐based devices. In this work, we investigate the potential of THz light to probe the viscoelastic behaviour of skin and derive age‐dependent parameters that could be used to quantify the effect of a variety of treatments and cosmetics. In turn, this technique can be utilised for simultaneous in vivo assessment of skin biomechanics and condition.

## 1 Introduction

### 1.1 Terahertz Light

The terahertz (THz) frequency band of the electromagnetic spectrum is defined as light with a frequency between 0.1 and 10 THz, where 1 THz is equal to $10^{12}$ Hz. Historically, developing practical methods of THz generation and detection had been difficult and research stalled, thus forming the so‐called ‘terahertz gap’ [1]. However, since bridging of this gap, THz has shown significant promise in a number of wide‐spanning applications. Of particular note is its use in biomedical diagnostics, where the high sensitivity of THz to biological water content has made it an encouraging tool for cancer detection [2, 3]. Additionally, THz has been investigated as a tool for in vivo sensing benign skin conditions such as eczema and psoriasis, and in evaluating the efficacy of transdermal pharmaceutical patches [4, 5, 6].

In vivo measurements require the THz system to be arranged in a reflection geometry, due to the high absorption of THz waves by water, in which an incident THz beam is directed onto the sample and the sample properties are inferred from the reflected THz signal. A schematic of this geometry is shown in Figure 1. A quartz imaging window is used to contact the skin surface. This ensures phase alignment at the interface and provides an impedance matching effect between the air and the skin, increasing the penetration of the incident THz beam into the skin. The generated THz light propagates through the quartz window into the skin, and the reflected electric field is recorded in the time domain, providing time‐of‐flight information analogous to an ultrasound A‐scan. The resulting temporal waveform is then Fourier transformed to obtain the complex frequency‐domain response of the sample.

**FIGURE 1:**
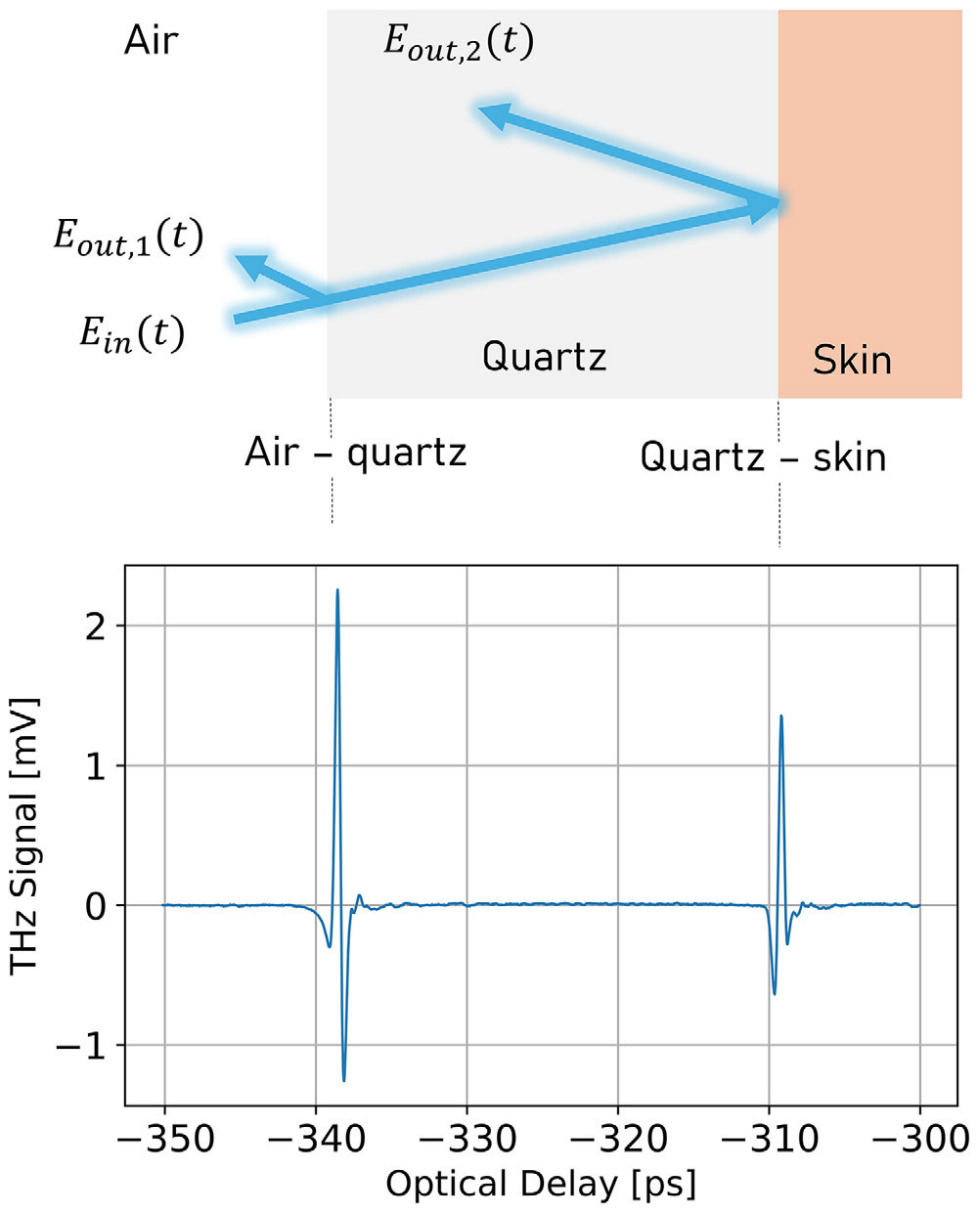
A schematic of a THz time‐domain spectroscopy (THz‐TDS) reflection geometry system used to measure the in vivo properties of skin. In the time‐domain, two reflections are detected: the first ($E_{\text{out},1}\left(t\right)$) from the air‐quartz interface, and the second ($E_{\text{out},2}\left(t\right)$) from the quartz‐skin interface.

### 1.2 THz Decay Curve and Skin Viscoelasticity

During in vivo measurements, it has been observed that the amplitude of the measured THz signal decreases as the contact time between the quartz window and the skin increases. The origin of this amplitude decay has previously been attributed to water accumulation within the outermost skin layers during occlusion [7]. Occlusion is when the skin surface is blocked by an impermeable barrier, in this case, the quartz window. However, literature suggests that this process occurs over a timescale ranging from tens of minutes to hours, rather than within the first few minutes of a measurement [8, 9]. Our previous work has shown that within the first few minutes of contact, the observed THz amplitude decay is primarily driven by improved THz coupling between the skin surface and the window. The skin surface is not perfectly flat, its microtopography contains a multitude of ridges, furrows, wrinkles and other asperities that can form microscopic air gaps beneath the quartz. Consequently, the incident THz light experiences an effective medium consisting of both air and skin in varying proportion. Initially, when the asperities are greatest, the proportion of air is large. However, during prolonged contact with the quartz, the asperities flatten and the contact between the quartz and the skin surface improves; the incident THz effectively experiences an increasing proportion of skin. This reduces the interfacial reflectivity at the quartz‐skin boundary, and drives a reduction in the detected THz signal [10].

Figure 2a shows an example of the observed decay in the amplitude of the THz signal. Specifically, the peak‐to‐peak (P2P) amplitude of the unprocessed reflected signal (the quartz‐skin reflection in Figure 1) is plotted as a function of contact time with the quartz. The inset shows the temporal evolution of the signal, highlighting the clear decrease in the P2P amplitude. It is also common to plot the P2P of the processed, deconvolved signal, known as the impulse function [11]. The decay curve exhibits bi‐exponential behaviour, characterised by an initial rapid decay followed by a slower, quasi‐linear regime. Similar bi‐exponential behaviour has also been observed in skin deformation studies that measure the mechanical response of skin under applied stress. The fast component of deformation is attributed to the elastic nature of skin, whereas the slower component is driven by viscous flow, which is intrinsically linked to the skin's water content. Since skin exhibits both elastic and viscous behaviour under applied stress, it is described as a viscoelastic material [12, 13].

**FIGURE 2:**
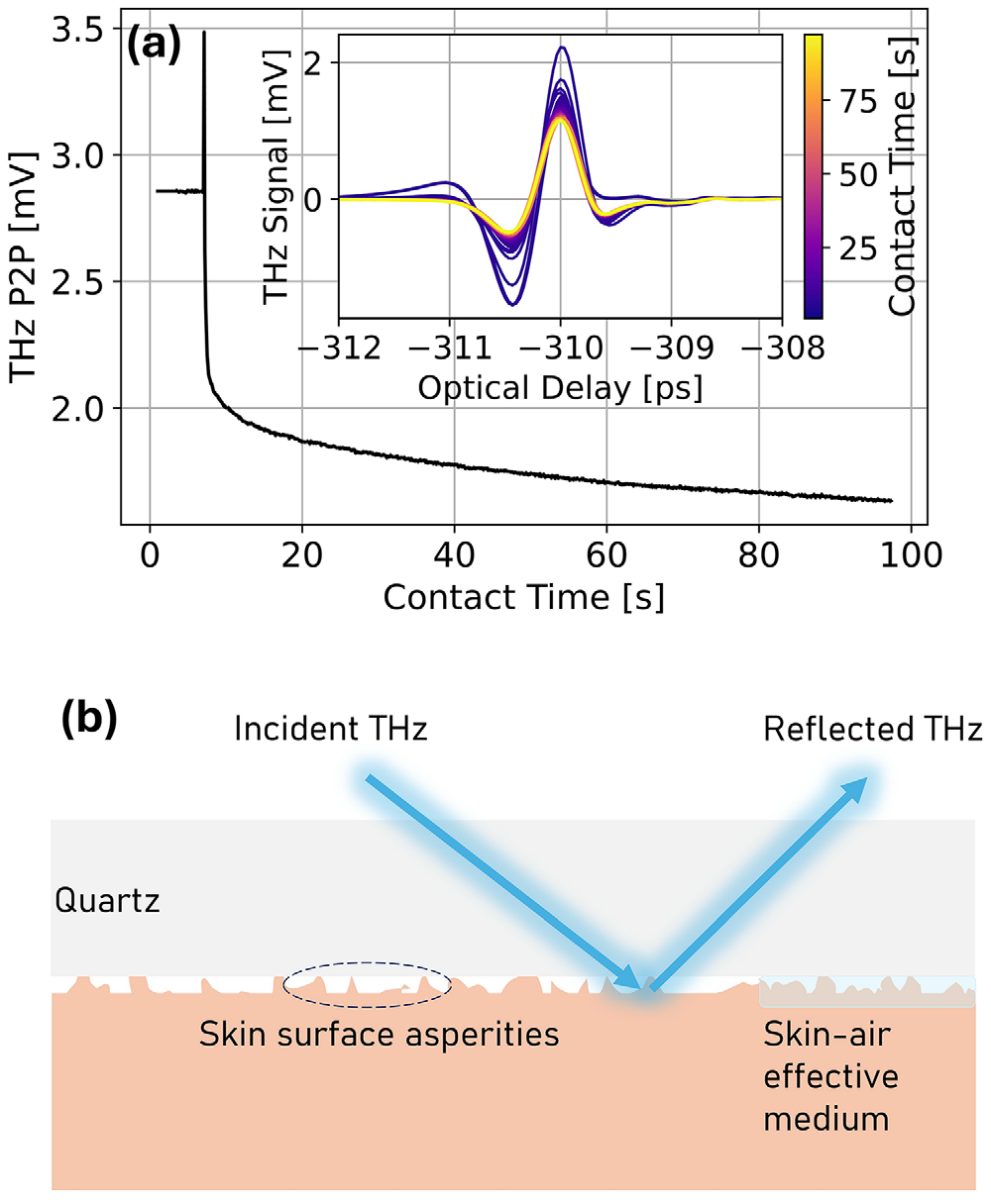
The THz decay curve and is primary driver. (a) An example of the THz decay curve in an in vivo measurement, represented by the decrease in the THz signal peak‐to‐peak (P2P) with contact time. The inset shows the change in the detected THz signal with contact time. (b) Prior to compression by the quartz window, the skin surface asperities are largest and the effective THz interaction region consists of a large proportion of air (diagram not to scale).

There are numerous studies investigating the viscoelastic properties of skin. Typically, these studies employ a Cutometer (or similar device) to measure skin deformation during suction cycles where the skin is loaded and unloaded with stress. A number of parameters can be calculated from the resulting deformation curves to gauge the viscoelastic properties and compare between participants [14, 15]. These parameters have also been suggested as biomarkers to aid diagnosis of skin conditions such as systemic sclerosis, and to monitor improvements in skin state during treatment [16, 17, 18]. In addition to suction‐based devices, alternative methods for assessing skin elasticity have also been explored. One such approach is elastography, in which mechanical wave propagation through tissue is measured and used to infer mechanical properties. For example, Saavedra et al. used a combination of ultrasound and crawling wave elastography to measure the in vivo viscoelastic properties of forearm and thigh skin [19]. However, these techniques do not provide deformation curves directly comparable to those obtained from suction‐based methods, and therefore require additional processing and modelling to extract equivalent viscoelastic parameters. Figure 3 shows a synthetically generated example of a skin deformation curve. The curve consists of two parts: a loading phase between 0 and 10s, and an unloading phase from 10 to 20s. During the loading phase, suction is applied and the skin deforms accordingly. During unloading, suction is removed and the skin deforms back towards its initial state. From this cycle, four parameters can be defined:

$U_{e}$
Initial elastic deformation
$U_{v}$
Viscous deformation
$U_{f}$
Total deformation during loading
$U_{r}$
Elastic retraction/recovery

**FIGURE 3:**
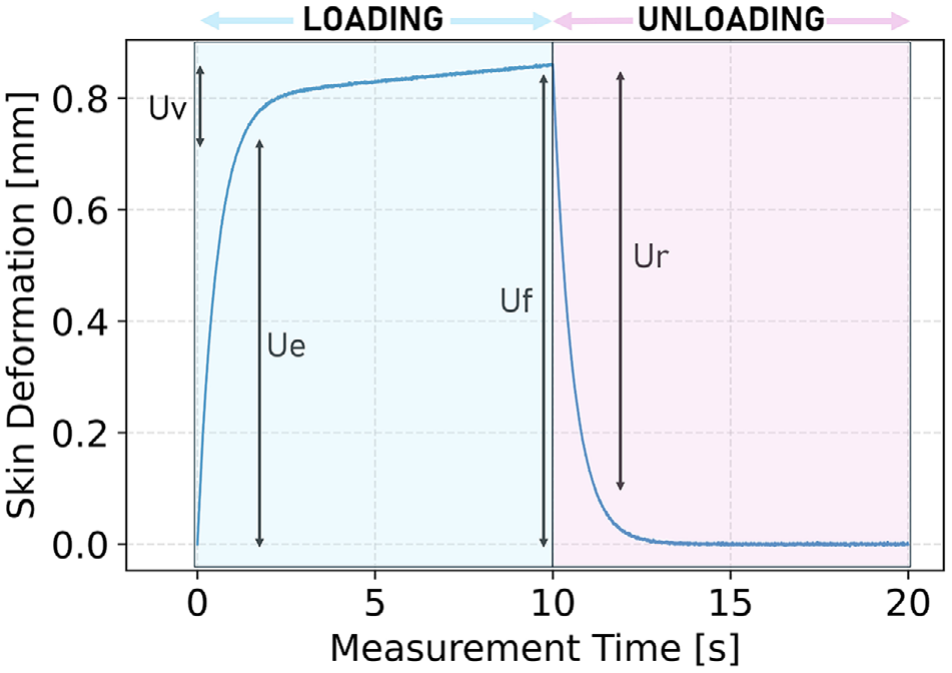
A synthetic example of the data obtained during a Cutometer measurement, showing the skin deformation and recovery during a loading and unloading phase. Ue is the initial elastic deformation, Uv is the viscoelastic deformation, Uf is the total deformation and Ur is the elastic recovery.

It is hypothesised that the viscoelastic deformation of the skin during contact by the quartz window defines the bi‐exponential nature of the THz decay curve. Thus, properties of the decay curve may act as proxies for the viscoelastic parameters defined by the skin deformation curve. This presents a unique benefit of THz to probe both dielectric and mechanical properties simultaneously, further enhancing its potential as a dermatological diagnostic tool. In this paper, we detail a number of experiments that may elucidate this hypothesis and discuss the potential of THz as a probe of skin viscoelasticity.

### 1.3 Poroviscoelasticity

As previously discussed, THz light is highly sensitive to variations in biological water content. Up to this point, we have described the mechanical behaviour of the skin as viscoelastic, however it is more accurately characterised as poroviscoelastic. This means viscoelastic deformability is partially governed by the flow of interstitial fluid throughout the skin's porous network. Under compression, hydraulic pressure within the tissue increases, generating radial and lateral pressure gradients that drive fluid redistribution [20]. As such, it is important to consider whether this water redistribution could affect the THz response.

Firstly, it should be highlighted that the THz decay curve shows a decrease in the THz signal amplitude with increasing contact time. This is contrary to what would be expected if fluid redistribution dominated ‐ the flow of water away from the compressed region would cause an increase in the reflected THz signal as the locally dehydrated tissue becomes less THz‐absorptive. Secondly, the penetration depth of THz is limited within biological tissue to approximately 50–200 μm (depending on frequency), meaning it probes the outermost layers of the skin—the stratum corneum (SC) and viable epidermis (VE) [21, 22]. It has previously been shown that fluid redistribution during loading is primarily concentrated within the dermis, owing to its high hydraulic permeability. The majority of water within the SC is bound to keratin, meaning it is not free to permeate through fluid channels ‐ water redistribution within the SC and VE is minimal [20]. As such, the effects of any water concentration changes within the penetration depth of the incident THz signal are negligible. The SC water content does, however, play an important role in modulating the viscoelastic behaviour. It has been shown that water uptake by the corneocytes within the SC has a plasticising effect on the keratin molecules controlling the viscoelastic behaviour of the SC [23]. Thus, whilst water content changes may not have a direct effect on the THz measurement, there is certainly an important indirect effect.

## 2 Theory

For in vivo THz measurements, the reflected signal from the sample is detected in the time‐domain as the electric‐field $E_{\text{meas}}\left(t\right)$. This measurement will contain a number of system artifacts, for example dispersion from optical elements or absorption by residual water vapour within the THz path. Hence, the measured signal is considered to be a convolution between the sample and system response:

(1) $$E_{\text{meas}}\left(t\right)=E_{\text{system}}\left(t\right)*h_{\text{sample}}\left(t\right)$$

where $E_{\text{meas}}\left(t\right)$ is the measured THz signal, $E_{\text{system}}\left(t\right)$ is the system response and $h_{\text{sample}}\left(t\right)$ is the impulse response of the sample.

Therefore, to isolate the sample response, it is necessary to perform a deconvolution wherein the system response is accounted for. This is done by measuring the system's response to a known sample (colloquially termed a reference measurement), for example air. Once this has been measured, deconvolution is performed as follows:

(2) $$M\left(\omega \right)=\frac{E_{\text{meas, sample}}\left(\omega \right)}{E_{\text{meas, ref}}\left(\omega \right)}$$

where M(ω) is the sample transfer function, $E_{\text{meas, sample}}\left(\omega \right)$ and $E_{\text{meas, ref}}\left(\omega \right)$ are the Fourier transformed sample and reference measurements. M(ω) contains the deconvolved sample response.

It is important to distinguish between t, the picosecond time scale over which the THz electric field exists and T, the measurement time (or contact time). In this work, $E_{\text{meas}}\left(t\right)$ is measured continuously while the force applied by the quartz window is varied. The measured signal therefore depends on the contact time, such that $E_{\text{meas}}\left(t\right)=E_{\text{meas}}\left(t,T\right)$. It follows that the transfer function, M(ω), also becomes contact time dependent, M(ω,T). For all studies presented in this paper, the term ‘THz decay curve’ refers to the evolution of M(ω,T). Further detail about deconvolution of THz signals can be found in [24, 25] and a review of the mathematical modelling of the skin's response at THz frequencies can be found in [26].

## 3 Materials and Methods

All THz measurements were performed using the PicoBot system developed in‐house at the University of Warwick. The system integrates a commercial THz spectrometer (Menlo TeraSmart) with a medical‐grade robot (KUKA LBR Med14) for THz sensing in a clinical setting. The system has both imaging and point measurement capabilities, and is discussed in detail in reference [27]. The quartz window consists of a circular z‐cut quartz with thickness 2mm and diameter 20mm. This window contacts the patient's skin and applies a precisely controlled compression force. The force is controlled using a force torque sensor that provides feedback to the KUKA joints via PID control, and maintains a stable force during the measurement period. The force torque sensor continually communicates with the KUKA arm, allowing the user to dynamically change the desired force throughout the measurement. In this way, compression force becomes a function of measurement time, T. THz signal and force acquisition are synchronised to ensure accurate temporal alignment. Further information about the PicoBot can be found in [28]. The PicoBot system is displayed in Figure 4a, with Figure 4b showing a schematic of the PicoBot probe containing the THz optics. Figure 4c shows the measurement time dependent force, F(T), and the THz signal, $E_{\text{out}}\left(t,T\right)$. Within the first few seconds, the compression force rises from 1 to 5N, indicating contact between the quartz window and the skin has been made. This is displayed in the THz signal; initially, until contact has been made with the skin, the quartz‐air reflection is measured. There is a sharp change in the THz signal once the quartz contacts the skin, and this reflection changes over time as the compression force is maintained.

**FIGURE 4:**
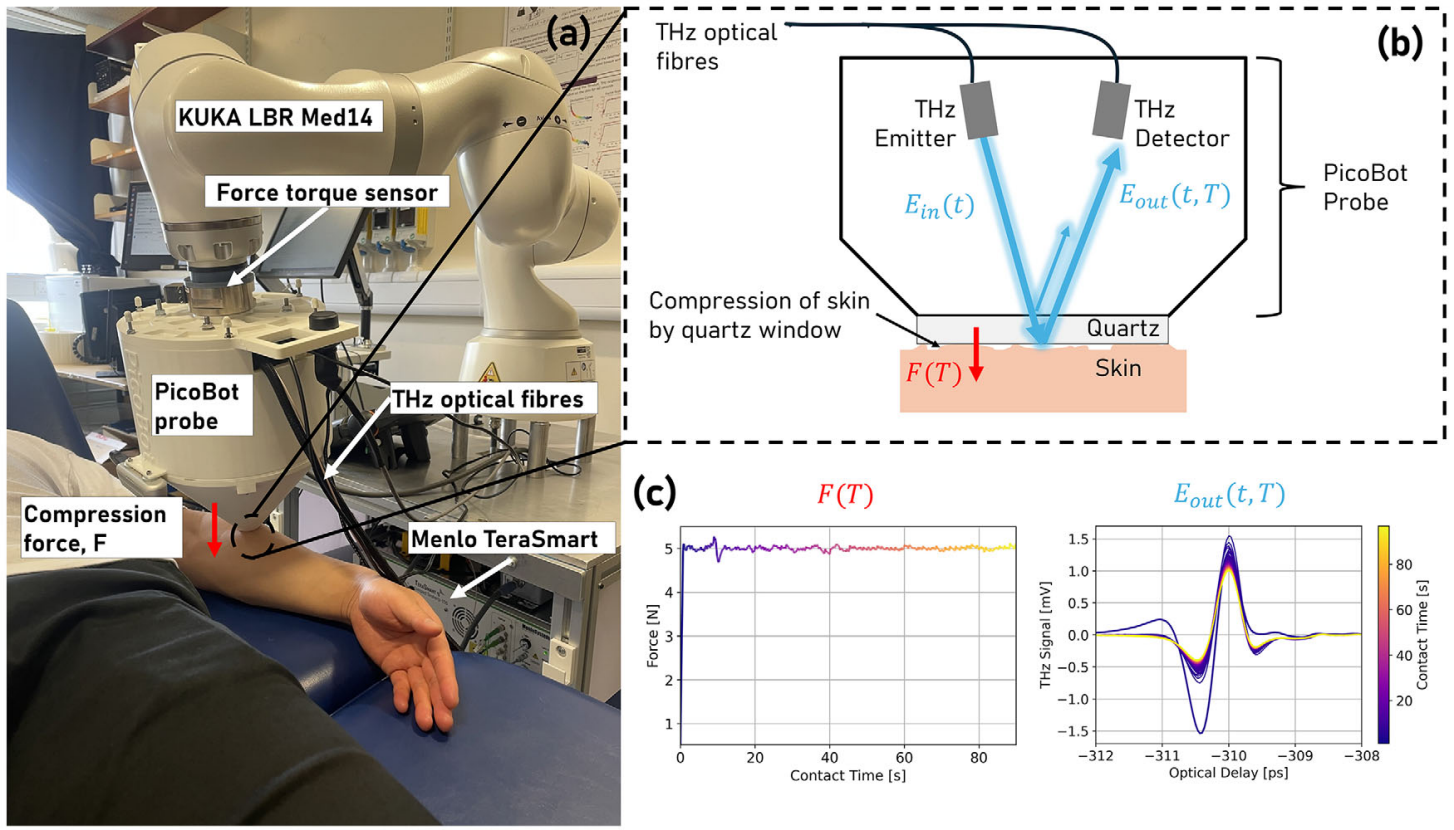
(a) A photograph of the PicoBot system being used to measure the THz response of the volar forearm. (b) A schematic of the PicoBot probe showing the fibre‐coupled THz detector and emitter, and the quartz window compressing the skin. The THz electric field, $E_{\text{out}}\left(t\right)$, is a function of measurement time, T. A force torque sensor is used to control and measure the compression force, F(T). (c) The compression force and THz response of skin during a 90 s measurement.

The THz path was purged with dry air to reduce the effects of water vapour. For repeatability, all measurements were taken on the volar forearm of the participant where the effects of photodamage or external influence are minimal. The data presented in Section 4.4 was collected as part of the TERABOTICS study at University Hospitals Coventry and Warwickshire (UHCW). Ethical approval was awarded by the Health Research Authority (HRA) and Health and Care Research Wales (HCRW) for the TERABOTICS study (IRAS project ID: 270335). All patients had given their informed consent prior to beginning any measurements. During the study a single‐point measurement was taken on the patient's volar forearm for 90 s at an applied compression force of 5N. During the measurement, THz acquisition was initialised prior to the quartz window contacting the skin. This is shown by the pre‐decay plateau in Figure 2a; the plateau is the peak‐to‐peak of the THz response to air, in other words, the reflection from the quartz‐air interface. Following the plateau, there is a spike in the THz signal caused by interference effects as the separation between the window and the skin surface decreases. Once the window has made contact with the skin, the THz signal begins to decay. To ensure comparability between measurements, each of the THz decay curves were aligned to the point at which the signal passes through the plateau value after the interference spike. This removes the interference spike which is a non‐skin related effect, and would lead to artifacts in our subsequent analysis. Signal acquisition was synchronised with the force measurement to ensure accurate temporal alignment. It should be noted that whilst participants recruited for the TERABOTICS study were undergoing excision of basal cell carcinoma, the measurements presented in this study were taken on the non‐lesional, clinically healthy volar forearm of each participant. Thus, the subsequent results represent the THz response of healthy skin only.

The data presented in Sections 4, 4.1, 4.2 were collected in‐lab at the University of Warwick. Appropriate informed consent was taken from each participant before measurements and ethical approval for the study was granted by the Biomedical and Scientific Research Ethics Committee (BSREC), application number BSREC 26/23‐24. Measurements were repeated three times on two participants. Each of the three repeats were taken on the same anatomical site. Measurements were taken 15 min apart to allow the skin to recover and mitigate against preconditioning effects. After 15 min of recovery, the THz response of skin returns to a baseline range. Owing to the intra‐participant variation in skin state, the data shown in Sections 4.2 and 4.3 were from a single participant.

The effects of environmental factors such as temperature and relative humidity were mitigated through the control of ambient conditions in both the laboratory at the University of Warwick and in theatre at UHCW. All participants were acclimatised to the ambient conditions prior to measurements. All research was performed in accordance with the relevant guidelines and regulations, including in accordance with the Declaration of Helsinki.

## 4 Results and Discussion

### 4.1 The Effect of Force

To begin with, the effect of applied force on the THz response is considered. In this study, the applied force was varied between 2 and 5N in 1N steps and the THz signal was measured for 90 s. For each force, the measurement was repeated three times and a standard error was calculated. The THz signal is represented by the magnitude of M averaged between 0.5 and 1 THz, $|\bar{M}\left(0.5\;-\;1\;\mathrm{THz}\right)|$. As shown in Figure 5a, a clear trend is observed in the THz decay curves: increasing force leads to an increase in the overall amplitude of the decay. A reduction in the standard error is also observed at higher applied forces. This may be explained by saturation of the skin's deformability at larger forces ‐ tissue deformation becomes more consistent and repeatable under compression. At lower forces, the response shows greater variation, likely reflecting increased sensitivity to local heterogeneity in the skin surface within the measurement region.

**FIGURE 5:**
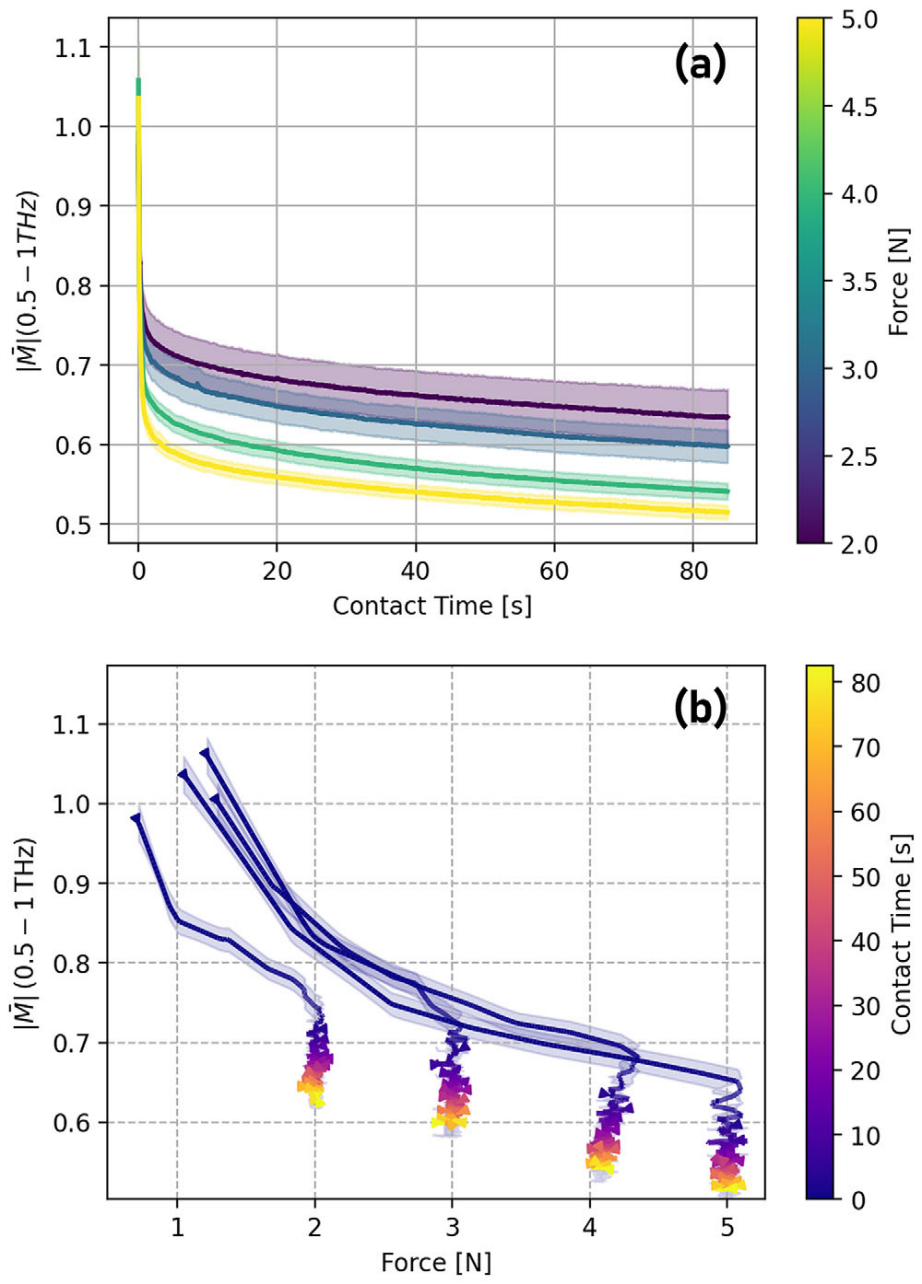
The effect of applied force on the THz response. (a) The variation in the THz decay curve with force, mean and standard error from three repeats is shown. (b) The force‐dependent trajectories of the THz signals.

Figure 5b shows the direct effect of the force variation on the THz response. The THz signal exhibits a force‐dependent path; there is a longer path length for greater forces representing a larger change in the THz signal. The THz signal for forces 3, 4 and 5N follow a similar path but with an end point dependent on the force. For 2N, however, the THz signal appears to follow a different path, perhaps indicating the insufficiency of 2N to induce comparable skin deformation. In the context of viscoelasticity, this result is entirely expected and makes intuitive sense ‐ greater force yields greater deformation [29].

### 4.2 Cyclic Loading

This experiment aims to replicate the cyclic application of suction from a Cutometer measurement. The applied contact force was cycled between 5 and 2N in 30s increments, while the reflected THz signal was measured continuously. Figure 6 shows the effect of the loading and unloading phases on $|\bar{M}\left(0.5\;-\;1\;\mathrm{THz}\right)|$. Similar to the results seen in Figures 5, and 6a shows a general negative trend with increasing contact time. This likely reflects viscoelastic creep of the skin under sustained loading, whereby the tissue continues to deform gradually under a constant applied force, potentially due to poroelastic fluid redistribution and/or structural relaxation. Reducing the force to 2N permits partial recovery of the skin, but not a full return to its initial state, as indicated by the increase in $|\bar{M}\left(0.5\;-\;1\;\mathrm{THz}\right)|$ during the unloading phase. The THz response exhibits a rapid initial recovery followed by a near‐plateau, indicative of a fast elastic recoil followed by a semi‐steady state during the reduced load phase. Subsequent loading causes a familiar bi‐exponential decay in $|\bar{M}\left(0.5\;-\;1\;\mathrm{THz}\right)|$, but with a significantly reduced elastic component. Over time, with sustained loading and unloading cycles, the skin deformation saturates and recovery during unloading phases is substantially impeded.

**FIGURE 6:**
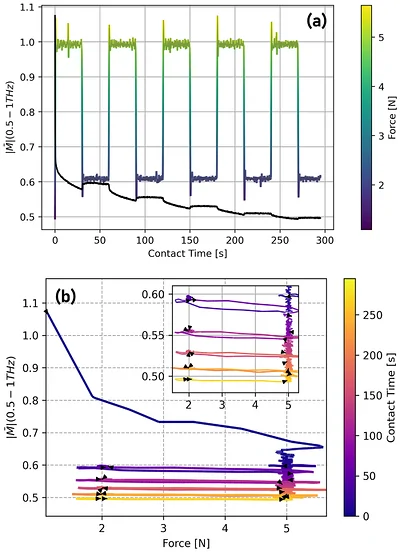
The effect of cyclic loading on the THz response. (a) The compression force cycle between 2 and 5N, with the corresponding THz decay curve shown by the change in $|\bar{M}\left(0.5\;-\;1\;\mathrm{THz}\right)|$. (b) The force‐dependent path of $|\bar{M}\left(0.5\;-\;1\;\mathrm{THz}\right)|$ showing the direct change during the loading and unloading phases, the colour indicates the contact time. The inset shows the hysteresis loops formed during the loading and unloading process.

Figure 6b shows the force‐dependent path of the THz signal; the colour represents the contact time in seconds. The inset highlights the paths following the initial drop as the force is cycled. There is a clear difference in the loading and unloading paths, which forms a loop during each cycle. In other words, the THz signal exhibits hysteretic behaviour with a clear decrease in the area of the hysteresis curve with subsequent cycles. This behaviour is expected for a viscoelastic material, where the strain for a given load is dependent on the mechanical history of the material. This is linked to mechanical preconditioning of the skin (sometimes known as the Mullins effect)—for later cycles, less stress is required to reach the same strain, hence the tissue is said to have become stress‐softened [30]. As loading is repeated, the tissue response becomes increasingly stabilised and the loading and unloading paths become more similar. This observed behaviour is in agreement with Cutometer measurements [15].

### 4.3 Force Ramps

Here, the THz signal was measured as the applied force was ramped in a stepwise manner from 3 to 6N in 0.5N steps every 30s. The resulting THz decay curve is shown in Figure 7a, with the inset highlighting the effect of the first two ramps. After the initial contact, each subsequent increase induces a progressively smaller change in the THz signal. However, these changes are still observable and appear bi‐exponential in nature—note the initial fast decay immediately after the force is increased. These changes are superimposed on the quasi‐linear decay driven by the continual creep deformation of the skin. As compression by the quartz window continues, the apparent elastic contribution to the response decreases and the deformation becomes increasingly dominated by viscous creep‐related processes. The THz coupling between the quartz window and skin surface continues to improve but at a much slower rate. Figure 7c highlights this effect; there is a clear decrease in the change of $|\bar{M}\left(0.5\;-\;1\;\mathrm{THz}\right)|$ with each subsequent increase in force.

**FIGURE 7:**
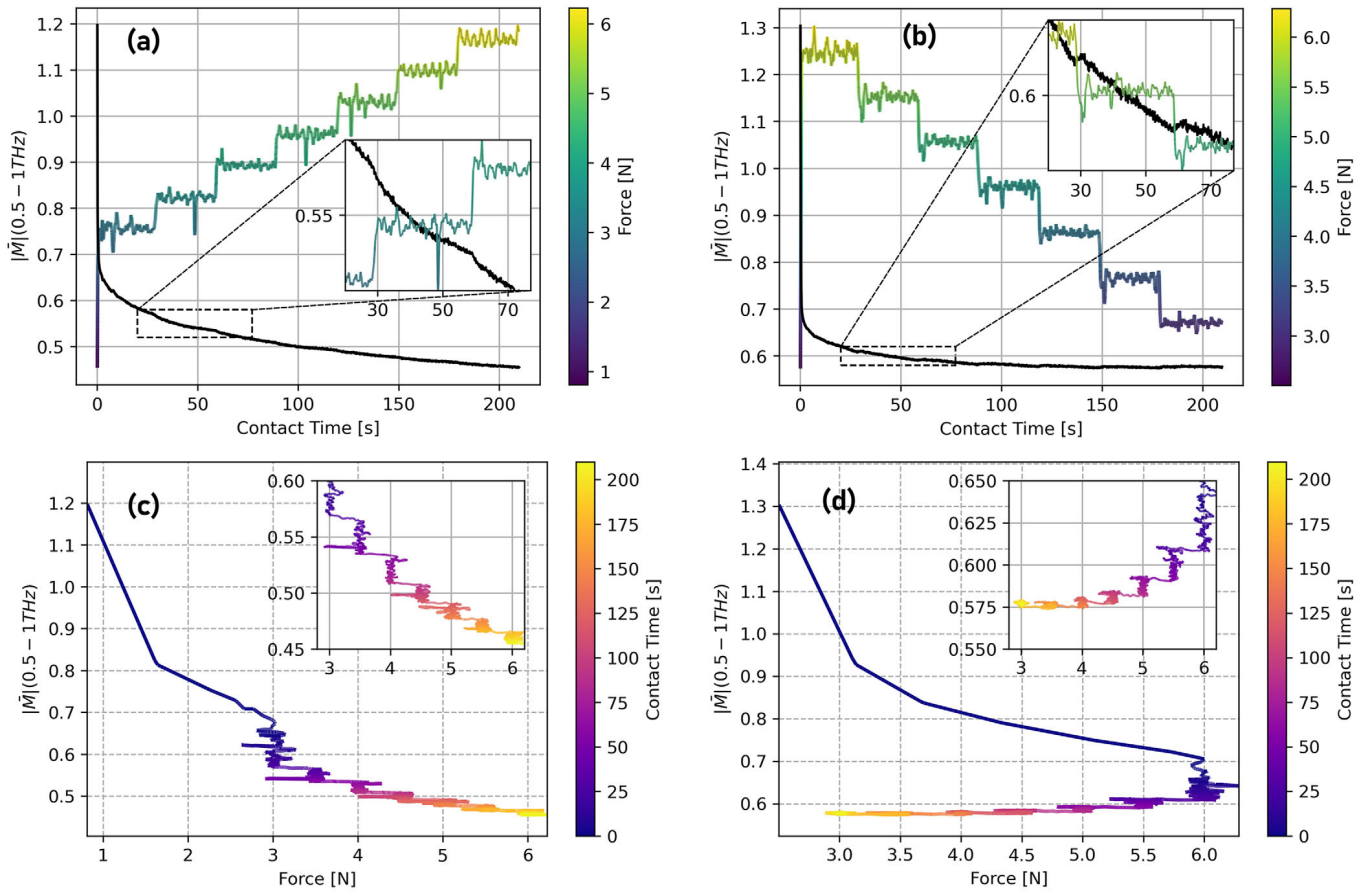
The effect of a stepwise force increase and decrease on the THz signal. (a) The effect of an increasing stepwise force on the THz signal. The inset highlights the stepped decay of the THz signal, showing the repeated fast decays after a period of constant force. (b) The effect of a decreasing stepwise force on the THz signal. The inset highlights the stepped recovery of the THz signal, showing the repeated recovery after a period of constant force. (c) The force‐dependent trajectory of the THz signal during a stepwise increase in the force. The colour scale represents the contact time. The inset highlights the stepped nature of the force. (d) The force‐dependent trajectory of the THz signal during a stepwise decrease in the force. The colour scale represents the contact time. The inset highlights the stepped nature of the force.

The effect of a decreasing stepwise force is shown in Figure 7b and d. There is a similar effect to the increasing stepwise force, but with the notable exception that during each force decrease there is a small increase in the THz signal. During this decrease, the skin surface asperities experience a small recovery which acts to reduce the THz coupling, thus leading to a slight increase in $|\bar{M}\left(0.5\;-\;1\;\mathrm{THz}\right)|$. The initial fast component of the decay is more prominent compared to the increasing force ramp, an expected result due to the greater starting force (6N compared to 3N). Throughout the measurement, there is still a quasi‐linear background decay as the skin continues to creep regardless of the force changes. Compared to the increasing force ramp, however, the total amount of creep deformation is reduced. This is explained by partial viscoelastic recovery and a lower net driving force for continued deformation under the reduced load. This difference highlights the history‐dependent response of the skin to loading; even though the cumulative stress is the same, the response is dependent on the order of loading.

### 4.4 Age‐Dependency

Since it is known that the properties of skin change with age, it is expected that such changes should be observable in the in vivo THz response. Figure 8 shows the age‐dependency of three different THz parameters: the initial fast decay ${\Delta |M|}_{1}$, the slower linear decay ${\Delta |M|}_{2}$, and the total decay ${\Delta |M|}_{\text{tot}}$. To assess each parameter's correlation with patient age, the Spearman correlation coefficient (ρ) has been calculated alongside its associated p‐value. The total number of patients included in this study was 37 (n = 37). These results are presented in Table 1.

**TABLE 1:** The Spearman correlation coefficient, ρ (*n*=37), and the *p*‐value for each parameter.

Parameter	ρ (*n*=37)	p‐value
${\Delta |M|}_{1}$	0.527	0.001^**^
${\Delta |M|}_{2}$	−0.300	0.071
${\Delta |M|}_{\text{tot}}$	0.380	0.021^**^
$\frac{{\Delta |M|}_{2}}{{\Delta |M|}_{1}}$	−0.467	0.004^**^

^**^ indicates a *p*‐value below 0.05.

**FIGURE 8:**
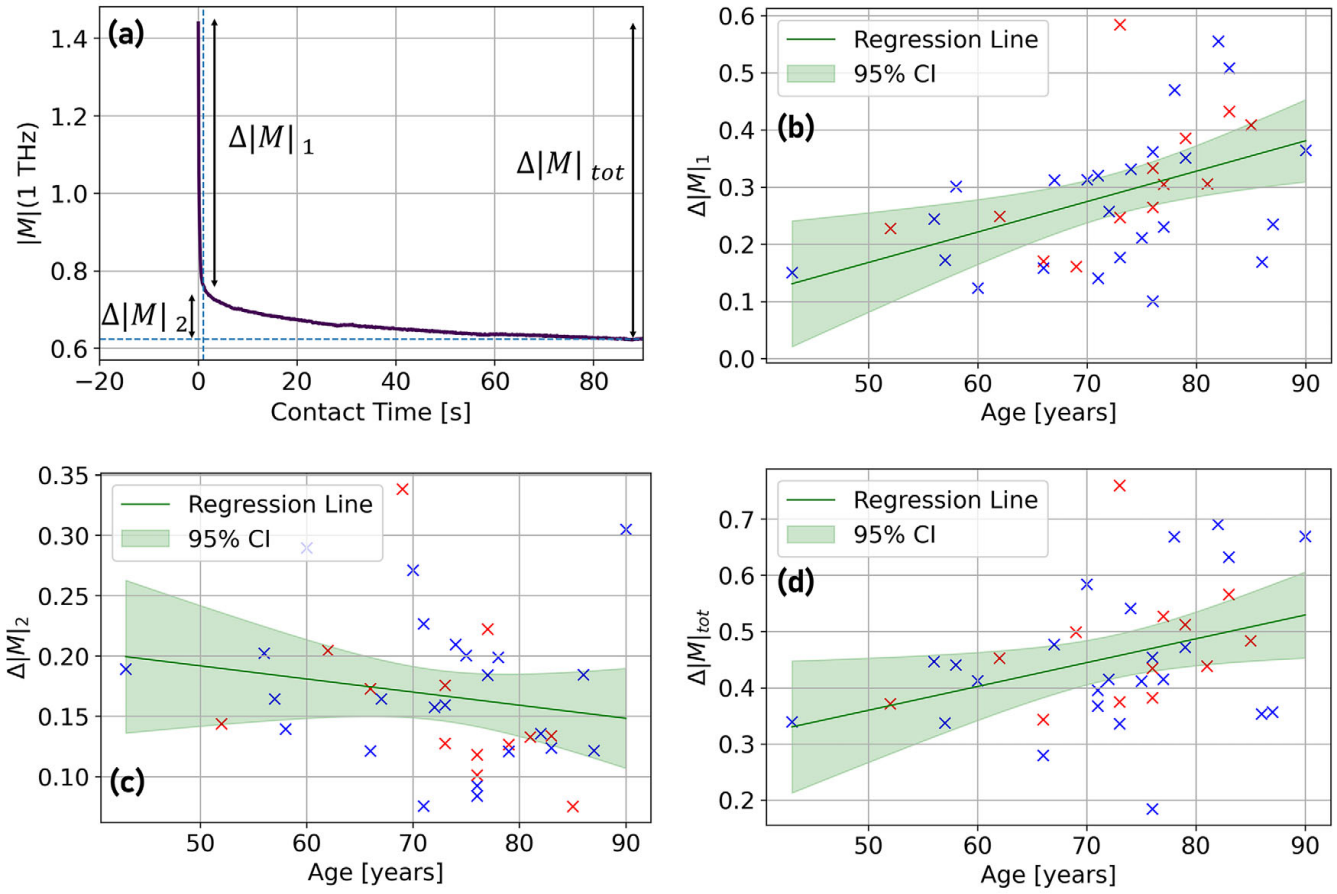
The age‐dependency of three M parameters which act as proxies for the skin viscoelasticity parameters shown in Figure 3. The best fit lines are calculated from a linear regression and the confidence intervals are calculated using bootstrapping of the Spearman correlation coefficient. The red markers represent female patients and the blue markers represent male patients. (a) The THz decay curve represented by the evolution of |M(1THz)| with contact time. From this curve, three parameters can be extracted: the initial fast change in |M(1THz)| (${\Delta |M|}_{1}$), the slow change in |M(1THz)| (${\Delta |M|}_{2}$), and the total change in |M(1THz)| over the measurement period (${\Delta |M|}_{\text{tot}}$). (b) The age‐dependency of ${\Delta |M|}_{1}$. (c) The age‐dependency of ${\Delta |M|}_{2}$. (d) The age‐dependency of ${\Delta |M|}_{\text{tot}}$.

The Spearman correlation coefficient quantifies the strength of the monotonic relationship between patient age and the selected THz parameter. It can range between −1 and +1, with ρ=0 implying no correlation and −1 or +1 a strictly monotonic relationship. It should be noted that the Spearman correlation coefficient is not equivalent to the Pearson correlation coefficient (r), which measures the strength of the linear correlation between two variables. In calculating the Spearman correlation coefficient, no assumption of linearity is made, and it simply measures the strength of monotonicity. This is of particular importance since it has previously been shown that the viscoelastic deformation parameters $U_{e}$, $U_{v}$ and $U_{f}$ do not necessarily exhibit a linear relationship with age. However, a positive monotonic relationship is expected for ages above 30 years. Interestingly, Agache et al. showed a negative monotonic relationship for age less than 30 years [31]. Our dataset is limited to an age range of 36–90 years, and thus we are unable to verify a negative monotonic relationship existing for ages below 30. Figure 8 shows the age‐dependency of the THz parameters, with the linear regression line used as a visual representation of the relationship. The suitability of the linear regression was confirmed by visual inspection of residuals and by comparison to a quadratic model which showed no statistically significant improvement in fitting.

As shown in Table 1 and in Figure 8b, the strongest correlation in age was observed for ${\Delta |M|}_{1}$. The Spearman correlation coefficient was 0.527 with an associated *p*‐value of 0.001, indicating a positive moderate‐to‐strong, and statistically significant, monotonic relationship between the initial, fast THz decay (${\Delta |M|}_{1}$) and patient age. Based on this result, in the context of skin viscoelasticity, the elastic deformability ($U_{e}$) of volar forearm skin is expected to increase with age. This is likely attributable to the decrease in skin elasticity measured in older people as skin collagen bundles degrade and become disorganised [32, 33]. The skin of older patients is less able to elastically resist compression, and consequently deforms more under the same applied force. It is noted that whilst ρ=0.527 is generally considered moderate‐to‐strong, it is clear from Figure 8a that age does not entirely explain the variance in ${\Delta |M|}_{1}$ and that outliers exist. This additional variation in ${\Delta |M|}_{1}$ may be explained by variability in lifestyle factors such as smoking, alcohol consumption or cosmetic use since these have been shown to impact skin health [34, 35, 36]. No statistically significant correlations between other tested participant characteristics were observed. These include sex, BMI, height, alcohol consumption and smoking status. With more data, however, correlations with alcohol and smoking status may be expected since these factors have been shown to influence skin state.

In addition to ${\Delta |M|}_{1}$, a positive monotonic correlation is also observed for ${\Delta |M|}_{\text{tot}}$, see Figure 8c. For this parameter, ρ=0.380 and the *p*‐value is 0.021. Hence, whilst the relationship between age and ${\Delta |M|}_{\text{tot}}$ is statistically significant, the strength of the correlation is considered moderate. Since ${\Delta |M|}_{\text{tot}}={\Delta |M|}_{1}+\Delta {\left|M\right|}_{2}$, the correlation of this parameter with age is largely driven by the correlation with ${\Delta |M|}_{1}$ as previously discussed. Conversely to the previous two parameters, ${\Delta |M|}_{2}$ exhibits a negative correlation with age with ρ=−0.300. However, the p‐value for this correlation is not statistically significant (p=0.071). In terms of viscoelasticity, Agache et al. also found no statistically significant correlation of $U_{v}$ with age. However, other research has shown a positive correlation between $U_{v}$ and age [37]. This highlights a potential limitation in using THz light as a probe of viscoelasticity. As the measurement duration increases and compression is sustained, the effect of viscoelasticity may no longer be isolated from the THz response. For example, dynamic processes occurring within the skin such as water accumulation or structural changes in the stratum corneum may influence the THz signal and lead to a divergence from a purely viscoelastic model of THz signal decay.

Finally, whilst not shown in the figure, the Spearman correlation coefficient for the ratio $\frac{{\Delta |M|}_{2}}{{\Delta |M|}_{1}}$ is −0.467 with a statistically significant p‐value of 0.004. Based on a purely viscoelastic model, this ratio is analogous to $\frac{U_{v}}{U_{e}}$. In contradiction to our result, Agache et al. found a positive correlation between this ratio and age. Our result is explained by the negative correlation observed for ${\Delta |M|}_{2}$ and the reciprocity of ${\Delta |M|}_{1}$ in the ratio. Also, the divergence of the ${\Delta |M|}_{2}$ parameter from its viscoelastic counterpart $U_{v}$, limits the practicality of the ratio.

The limitations of our dataset should also be considered. The entire dataset was collected from participants who were part of the TERABOTICS study and were undergoing excision of a suspected basal cell carcinoma (BCC). Due to the nature of the disease, these participants were all Caucasian and had a mean age of 72.4 ± 10.2 years (mean ± SD). Changes to the mechanical properties of the skin have been observed between races, and thus individual differences in the THz parameters may be identified [38]. However, the trends observed in this study are expected to generalise across a broader population since age‐related collagen degradation is universal. Further work should aim to extend the measured dataset and validate this expectation. Additionally, further work should increase the dataset to include a wider range of ages, particularly under 50 years. Indeed, Agache et al. observed a non‐monotonic relationship between age and the viscoelastic parameters for ages below 30 years [31]. Since all measurements were taken on the non‐lesional, healthy volar forearm of each participant, the presence of suspected BCC is not expected to affect the results since it is localised to the lesional area.

## 5 Conclusion

This work details an interesting proof‐of‐principle, wherein THz decay curve parameters may be used as proxies for quantifying the viscoelastic properties of skin. In doing so, the ‘skin age’ of a participant can be estimated based on the correlation of said parameters with age, offering useful cosmetic and dermatological information. It is also important to highlight the water sensitivity of THz, and thus the capability to simultaneously probe dynamic subcutaneous variations in water content. For example, based on a number of in vivo models, we can extract hydration‐related parameters from the THz response. Of course, the viscoelasticity is also intrinsically linked to skin hydration and the plasticising effect of water, thus the use of THz may reveal interesting interplay between the two properties. Further work should aim to better quantify the effect of skin hydration on the in vivo THz response and to understand its effect on the properties of the THz decay curve. The quasi‐viscoelastic parameters extracted from the THz decay curve should also be compared to parameters obtained from an associated Cutometer measurement, to identify the exact relationship between the two.

## Conflicts of Interest

The authors declare no potential conflict of interests.

## Data Availability

The data that support the findings of this study are openly available in Figshare at https://doi.org/10.6084/m9.figshare.32187120.
